# Predicting the effects of environment and management on cotton fibre growth and quality: a functional–structural plant modelling approach

**DOI:** 10.1093/aobpla/plu040

**Published:** 2014-07-09

**Authors:** Xuejiao Wang, Lizhen Zhang, Jochem B. Evers, Lili Mao, Shoujun Wei, Xuebiao Pan, Xinhua Zhao, Wopke van der Werf, Zhaohu Li

**Affiliations:** 1 College of Resources and Environmental Sciences, China Agricultural University, Beijing, China; 2 Centre for Crop Systems Analysis, Wageningen University, Wageningen, The Netherlands; 3 College of Agronomy and Biotechnology, China Agricultural University, Beijing, China; 4 Institute of Cotton Research of Chinese Academy of Agricultural Sciences, Key Laboratory of Cotton Genetic Improvement, Anyang, Henan, China

**Keywords:** Cotton (*Gossypium hirsutum*), fibre length, fibre strength, functional–structural plant model (FSPM), growth and development, micronaire, simulation model.

## Abstract

In general, the quality of fruits depends on local conditions experienced by the fruit during its development. In cotton, fruit quality, and more specifically the quality of the fibre in the fruit, depends on interactions between fruit position in the plant architecture, temperature and agronomic practices, such as sowing time, mulching with plastic film and topping of the plant's main stem and branches. To quantify this response of cotton fibre quality to environment and management, we developed a simulation model of cotton growth and development, CottonXL. Simulation of cotton fibre quality (strength, length and micronaire) was implemented at the level of each individual fruit, in relation to thermal time (represented by physiological age of the fruit) and prevailing temperature during development of each fruit. Field experiments were conducted in China in 2007 to determine model parameters, and independent data on cotton fibre quality in three cotton producing regions in China were used for model validation. Simulated values for fibre quality closely corresponded to experimental data. Scenario studies simulating a range of management practices predicted that delaying topping times can significantly decrease fibre quality, while sowing date and film mulching had no significant effect. We conclude that CottonXL may be used to explore options for optimizing cotton fibre quality by matching cotton management to the environment, taking into account responses at the level of individual fruits. The model may be used at plant, crop and regional levels to address climate and land-use change scenarios.

## Abstract (precis)

Fruit quality and more specifically quality of the fiber in the fruit of cotton, depends on interactions between fruit position in the plant architecture, temperature and agronomical practices, such as sowing time, mulching with plastic film, and topping of the plant's main stem and branches. A functional and structural cotton model CottonXL for fiber quality (strength, length and micronaire) was implemented at the level of each individual fruit in relation to thermal time for optimizing cotton fiber quality by matching cotton management to the environment. The model may be used to address climate and land use change scenarios.

## Introduction

In many plant species, fruit quality ultimately depends on local conditions experienced by the fruit during its development. In cotton (*Gossypium hirsutum*), fruit quality and more specifically the quality of the fibre is the result of an interaction between genetic (Richard *et al.* 2006), environmental (Liakatas *et al.* 1998; Zhao and Oosterhuis 2000; Pettigrew 2001; Yeates *et al.* 2010) and management factors (Pettigrew and Adamczyk 2006; Read *et al.* 2006; Girma *et al.* 2007), and is determined by the position of, and the resources captured by each fruit. Fibre quality of the crop also depends on agronomic practices such as sowing date, shoot topping (removal of main stem and branch apices) and film mulching. Fibre quality is a key factor determining fibre price and quality of cotton textile products. Fibre quality is characterized by fibre length (mm), strength (cN tex^−1^) and micronaire (unitless), and the textile industry has a preference for long and strong fibres of moderate micronaire for producing high-quality yarns (Long *et al.* 2010). Fibre strength is measured by assessing the force at which a standard fibre sample will break into units of cN (centiNewton) per tex (ICC standard). Micronaire measures fibre fineness and maturity (Saville 2004; Ren *et al.* 2013). Although previous studies addressed the formation of fibre, fibre quality of a plant or population at the field level as a whole remains difficult to predict.

**Figure 1.**
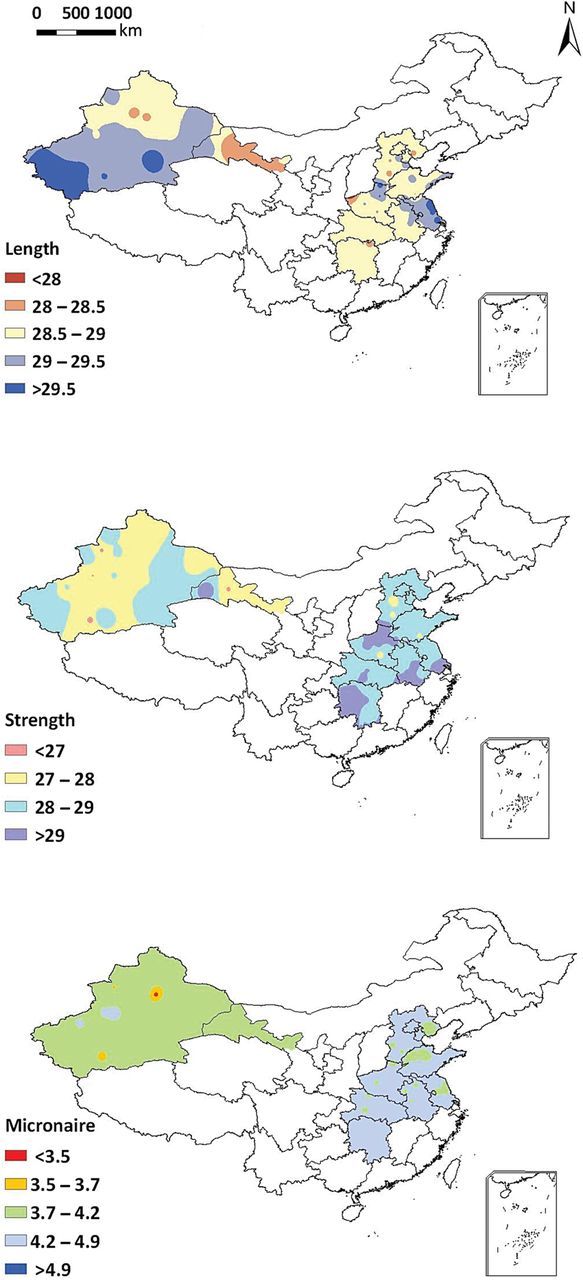
Cotton fibre length, strength and micronaire in the three major cotton producing regions in China. The fibre length (mm), strength (cN tex^−1^, 1 cN = 1.02 g) and micronaire are mean values measured with HVI900 according to ICC standard (data from the national-wide survey in China, averaged from 2006 to 2011).

Fibre quality of a cultivar may differ substantially between different environments (Bradow *et al.* 1997; Davidonis *et al.* 2004; Campbell and Jones 2005). In China, there are considerable differences in the quality of cotton fibre between the three most important cotton growing regions, Xinjiang, the Yellow River basin and the Yangtze River basin (Fig. 1). These quality differences are associated with meteorological differences (e.g. temperature, light and precipitation) and soil factors (e.g. texture and fertility). Especially, temperature is a major determinant of fibre quality due to its effect on fibre elongation and secondary cell wall thickening. Low temperatures result in decreased fibre length (Zheng *et al.* 2012). The cotton boll maturation period is prolonged with the progression of the growing season due to decreasing temperatures (Anderson and Kerr 1938). The maximum elongation rate and duration of fibre growth depend on the variety and on environmental factors (Gipson and Ray 1969). Optimum temperature for fibre elongation is slightly lower than optimum temperature for boll development (Pettigrew 2001). Decreasing the night-time temperature prolongs fibre elongation duration and decreases elongation rate (Gipson and Ray 1969). However, effect of temperature on secondary cell wall thickening is debated (Gipson and Ray 1969; Zhao *et al.* 2013). Fibre quality is also reduced by drought (Nehra and Yadav 2013) and insufficient nitrogen (N) supply (Zhao *et al.* 2012).

Models have been widely developed and used to explore and predict relationships between fibre quality and environmental factors. Statistical models have been developed based on regressions between fibre quality indices and temperature or radiation (Heskenth and Low 1968; Wanjura and Barker 1985; Sequeira *et al.* 1994). Also, a process-based model simulating fibre quality for individual fruits in response to temperature and N supply has been developed (Zhao *et al.* 2012). Models with complex parameters often require local calibration before they are applied at different regions (Baker *et al.* 1983; Hearn 1994). A generic cotton model SUCROS-Cotton (Zhang *et al.* 2008) simulates cotton growth and development based on a physiological day approach (Soltani *et al.* 2006), which is the days required for crop growth and development under optimal temperature. However, these models consider the fruit only and therefore cannot be applied to the analysis of fibre quality at plant or crop level because a cotton plant carries multiple fruits of different ages and developmental stages, with differences in accumulated thermal time in relation to position on the plant and in the canopy. The factors that affect fibre quality (notably temperature) are thus different for each single fruit, which needs to be taken into consideration for proper quantification of fibre quality. The models reported until now are not qualified for predicting fibre quality difference at both organ and crop levels. A model with detailed representation of the age and stage distribution of the fruit on the plant and in the canopy is therefore needed.

Scaling up fibre quality of individual fruits as determined by their local conditions to the plant and canopy levels is possible using the methodology of functional–structural plant modelling (FSPM) (Vos *et al.* 2010). In FSPM plant physiological processes and environmental factors can be integrated in representations of plant development. This enables simulation and analysis of the interactions between plant architecture and functioning. Functional–structural plant modelling is a versatile tool to simulate plant development, reproducing plant topology and geometry in response to environmental and internal factors. Linking physiological and architectural models, virtual cotton models have been developed to assist in interpretation of the plant maps (distribution of organs within the plant) for helping crop management (Jallas *et al.* 2000; Hanan and Hearn 2003). However, these models do not include fibre quality and the effects of manipulation of plant structure, such as branch topping and removal of vegetative branches. An FSP model of cotton development, CottonXL, has been designed and calibrated to simulate development of leaf and fruits (square, flower and boll), plant height and branching (Gu *et al.* 2014). Crop development in CottonXL is driven by thermal time, population density, application of growth regulator mepiquat chloride (MC) and topping of the main stem and branches. This model allows to simulate cotton fibre development in relation to the temporal and spatial distribution of retained fruits.

The main objective of this study was to assess cotton fibre quality in response to temperature regimes and agronomic practices (sowing date, main stem and branch topping, film mulching), in order to understand how the fibre quality of individual bolls is determined by the environmental conditions experienced by each boll during boll development, in relation to overall plant architectural development, and thus how high-quality cotton production can be optimized. For this purpose, routines calculating fibre quality were integrated in CottonXL using concepts from a process-based cotton model SUCROS-Cotton (Zhang *et al.* 2008). This yielded a model that simulates fibre quality determined by the conditions experienced by the individual fruits during their development on the plant. The model was subsequently calibrated and validated for fibre quality using experimental data. Finally, scenario studies were carried out to assess the effect of sowing date, main stem and branch topping, and film mulching.

## Methods

### Field experiments

Field experiments were conducted to determine fibre quality of individual fruits in Anyang (114°13′E, 36°04′N), Henan, China in 2007. The experiments included two cotton (*Gossypium hirsutum*) cultivars (Kemian 1 and NuCOTN 33B) grown under nonlimiting N supply (480 kg total N ha^−1^). Kemian 1 is a local bred Bt cotton which is popularly used in south China, and NuCOTN 33B is a Bt cotton from Monsanto, USA, which is popularly used in north China. The two cultivars both are middle matured cotton. The soil type was a sandy loam with 14.7 g kg^−1^ of organic matter content, 9.4 mg kg^−1^ of total N, 23.6 mg kg^−1^ of available P and 71.3 mg kg^−1^ of available K. Cotton was sown on 25 April. Half of the N fertilizer was applied before sowing and the rest was top-dressed on 15 July. Potassium chloride of 225 kg ha^−1^ and super phosphate of 750 kg ha^−1^ were applied basally. The experiments were designed as randomized complete blocks with three replicates. Each plot was 54 m^2^ (9 × 6 m) with 0.75 m row spacing and 0.25 m plant distance. All plants at middle four rows in each plot were observed per plot. This experiment partially overlapped with the one described in Zhao *et al.* (2012) and gathered independent experimental data for unlimited water and N treatments at Anyang in 2007.

Weather data were obtained from the national meteorological information centre (www.cma.gov.cn/2011qxfw/2011qsjcx). Accumulated thermal time, total precipitation and sunshine hours during the cotton growing season (April to October) at the sites for calibration, validation and scenario analyses are shown in Table 1. The detailed time courses of minimum and maximum temperatures during a crop season for all testing sites are given in **Supporting Information**.

**Table 1.** Weather condition in sites for calibration, validation and scenario analysis. ^a^Cumulative temperature, sunshine hours and rainfall from 1 April to 31 October are given for the year 2007 for the Anyang site. Values for other sites are based on averages from 2006 to 2011.

Region	Site	Above 12 °C temperature sum (degree days)	Sunshine (h)	Precipitation (mm)
Yellow River region	Anyang^a^	4561	1147	403
	Huimin	4518	1498	521
	Suiyang	4679	1149	639
	Xinye	4889	1027	682
Yangtze River region	Yueyang	5245	1273	861
	Rudong	4894	1157	946
	Wangjiang	5236	1113	1008
	Jiangling	4568	1327	665
Northwest region	Dunhuang	3994	2413	36
	Yumen	3225	2030	61
	Aksu	4335	1843	59
	Shihezi	3892	2052	117

### Data for model parameterization

Observations on fibre length, strength and micronaire of individual fruits of the same developmental age for both cultivars combined, under sufficient water and nutrient supply, were used to determine potential fibre quality for individual fruits. To collect these data, fruits at the first and second node positions of each fruit branch on randomly selected cotton plants were tagged on 18 and 27 July, and 15 August. The age of each tagged fruit, expressed in days after squaring (a square, or flower bud, was counted when the bud was larger than 3 mm in diameter), was recorded. Twenty tagged fruits or open bolls were picked at an age of 28, 33, 40, 47, 54, 61, 68 and 75 days after squaring. The sampled fruits were used for measurement of cotton fibre quality. The immature cotton fibres were hand-ginned and allowed to dry at room temperature. The fibres for immature or mature bolls were prepared before the measurements under the same humidity and temperature conditions. Fibre length was measured using the water washing method (Thaker *et al.* 1989) for young bolls (<53 days after squaring), and with a Y-146 cotton fibre photodometer (Taicang Electron Apparatus Co. Ltd, China) for older bolls (Zhao *et al.* 2012). Cotton fibre strength and micronaire were determined with an Uster High Volume Instrument 900 (HVI 900; Uster Technologies Ltd, Switzerland) fibre testing system (Anonymous 2001) at the Cotton Quality Supervision, Inspection, and Testing Center of China Ministry of Agriculture, Anyang, China. Since fibre strength and micronaire can only be measured for relatively mature bolls, fewer data on strength and micronaire were available compared with fibre length.

To determine the effect of temperatures on fibre length, strength and micronaire, previously published data (Ma *et al.* 2006) were reanalysed. The experiments were conducted in Nanjing 118°47′E, 32°03′N), Anyang, Baoding (115°47′E, 39°05′N) and Shihezi (86°01′E, 44°26′N) in 2002–03. Three different sowing dates in each site and year were compared with three replicates. All flowers at the third fruit branch in Shihezi and the fifth fruit branch in the other sites in each plot were labelled and harvested to measure fibre length, strength and micronaire. The relationship between the relative fibre quality (actual fibre quality divided by maximum fibre quality) for all individual fruits and temperature factors (daily average, minimum, maximum and the difference between minimum and maximum) was regressed using appropriate quadratic or linear models (see the Results section).

### Data for model validation

**Figure 2.**
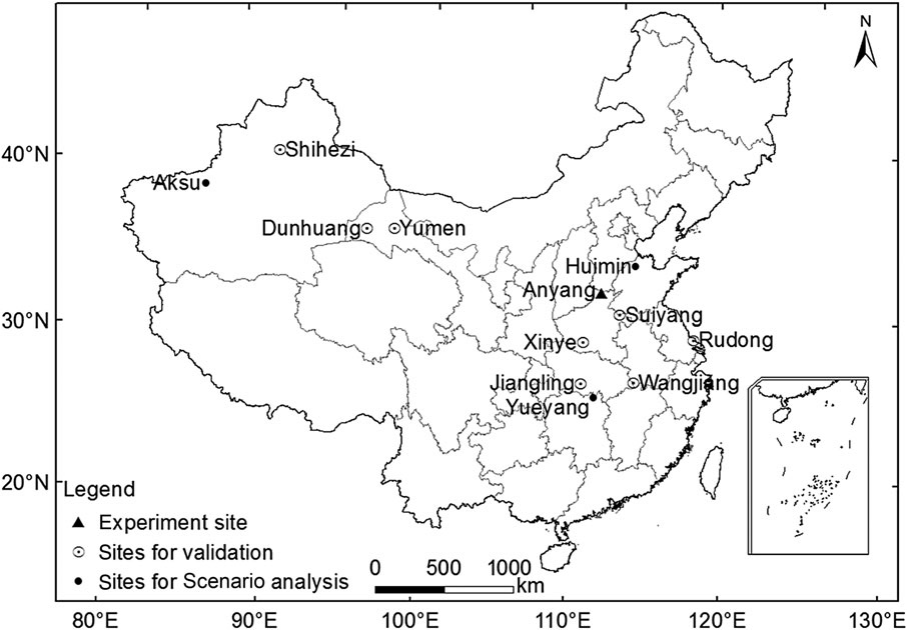
Locations of study sites for calibration, validation and scenario analyses.

Cotton fibre quality data (fibre length, strength and micronaire), measured with an HVI 900, were assembled from a nation-wide survey in three cotton producing regions in China from 2006 to 2011. Data of 82 sites located in the three major cotton producing regions, averaged from 2006 to 2011, were used to map the geographic distribution of fibre quality parameters in China (Fig. 1). Data from 11 typical sites, which represented three major cotton producing regions (4 sites for Xinjiang, 3 sites for Yellow River region and 4 sites for Yangtze River region) from 2006 to 2011 (Fig. 2) were used to validate the model.

### Development of an FSP cotton model for fibre quality

Cotton development and fibre quality were simulated using CottonXL (Gu *et al.* 2014), which is based on the principles of FSPM (Vos *et al.* 2010), and is developed using the GroIMP platform (available for free on www.sourceforge.net/projects/groimp) (Hemmerling *et al.* 2008).

#### Physiological days

Crop development in CottonXL is driven by thermal time, expressed as physiological days, i.e. the number of days required for growth and development of an organ under optimal temperature (Zhang *et al.* 2008). Physiological days take the effects of both base and optimal temperatures into account. The physiological age of an organ is the sum of daily temperature effect (*E*(*T*)):

(1) $$E(T)=\frac{T-T_{b}}{T_{o}-T_{b}}$$

where *T* is the daily mean temperature calculated by averaging minimum and maximum temperatures. *T*_b_ is the base temperature and *T*_o_ is the optimal temperature. Temperatures between 21.9 and 29.7 °C are optimal for cotton boll development, which determines fibre quality; however, temperatures as low as 15 °C still do not arrest cotton boll development (Gipson and Ray 1969; Dusserre *et al.* 2002). Therefore we used 12 °C for *T*_b_ and 25 °C for *T*_o_ (Zhang *et al.* 2008). Additionally, the daily *E*(*T*) was set to zero when *T* was smaller than *T*_b_ and to 1 when *T* was greater than *T*_o_. The effect of film cover on physiological age was simulated by adding an increment of air temperature estimated based on soil temperature increased by plastic film and crop response (Zhang *et al.* 2008).

#### Simulation of plant structure and organs

**Figure 3.**
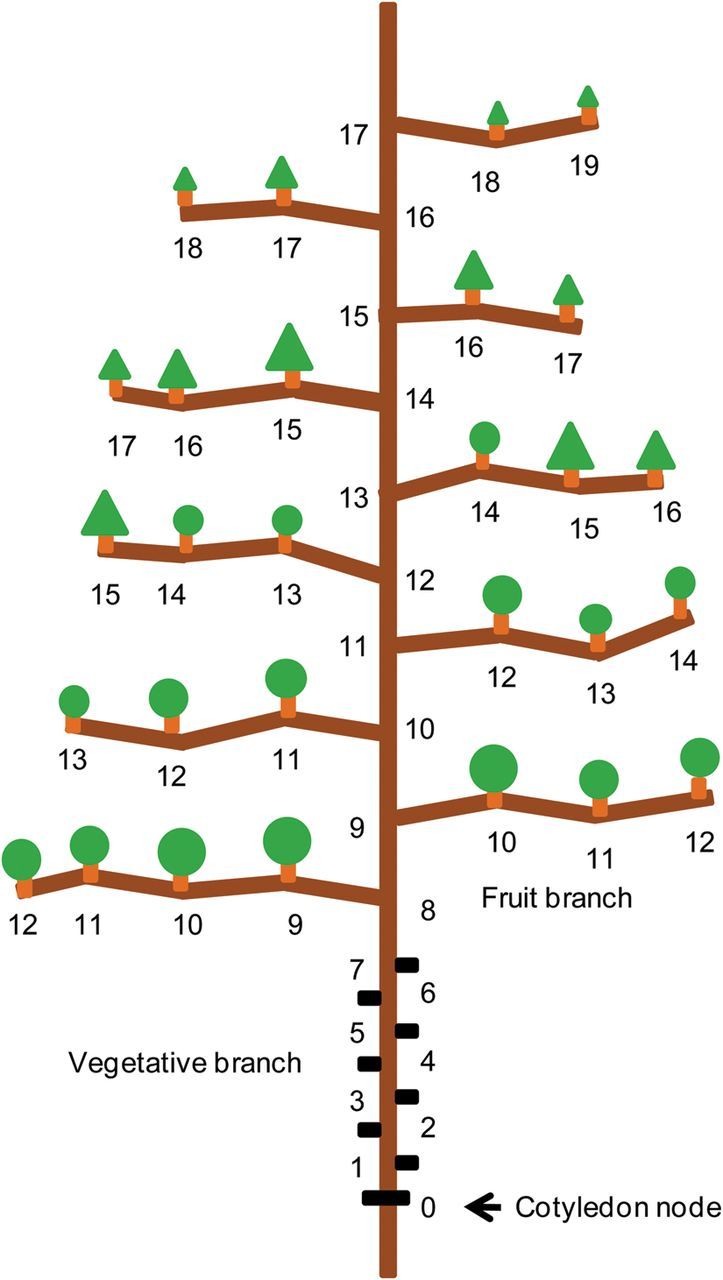
Cumulative phytomer number of fruits in cotton plants, counted from the cotyledon node.

CottonXL simulates development of cotton plant architecture at the level of the phytomer (Fig. 3) and its organs, explicitly taking into account organ appearance, extension, final size, location and orientation. The rate of organ appearance is determined by the plastochron. The value of the plastochron is fixed throughout plant development and expressed in thermal time. Organ development is calculated based on organ age, which itself is expressed in thermal time since organ appearance. Organs that have the same cumulative phytomer number (CPN, the phytomer rank of a fruit counted from the cotyledon node; Fig. 3) appear simultaneously and therefore have the same age. In the model, new phytomers appear at a plastochron of 3–3.5 physiological days. The value of the plastochron is a function of population density and the application of plant growth regulator MC. Topping of main stem and branches was simulated by removal of the apex and the top two phytomers (according to farmer's practice), resulting in termination of the appearance of new phytomers. Parameters for temperature-dependent growth and development rates of organs, fruit formation and abscission, and the effect of film mulching were taken from a process-based cotton growth model SUCROS-Cotton (Zhang *et al.* 2008). Fruits (square, flower and boll) appeared on each phytomer on fruiting branches. The fruit abscission rate depended on fruit age (Zhang *et al.* 2008), expressed in physiological days, which allows comparison of all the fruits at different positions. A full account of model parameterization can be found in Gu *et al.* (2014).

**Figure 4.**
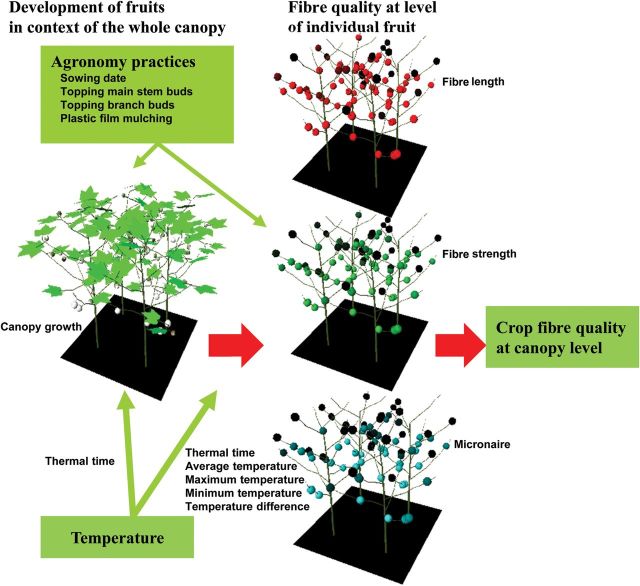
Illustration of the model concepts. At the level of the fruit, fibre quality (length, strength and micronaire) is calculated as determined by temperature (daily average, maximum, minimum and difference between maximum and minimum temperatures). Whole plant development is also driven by temperature, and is further determined by sowing date, topping and soil film cover. Output of the model includes distribution of cotton boll development within the plant and over time, as well as fibre quality of all individual bolls. Fibre quality aspects of length, strength and micronaire, with colour intensity represents the value (dark = low, bright = high).

We incorporated routines for the simulation of fibre quality in relation to temperature and individual fruit age in CottonXL. Fibre length, strength and micronaire of all individual bolls growing on the plant throughout the season were calculated on a daily basis taking into account the physiological time, the prevailing temperature and agronomic practices under sufficient water and N supply (Fig. 4).

#### Simulation of fibre quality

We assumed that fibre quality can be modelled as a growth process with physiological age after the emergence of a square. Accordingly, we described a potential fibre quality index under optimal conditions as a logistic equation against physiological days:

(2) $$Q_{i}=\frac{Q_{\max,i}}{1+{\text{e}}^{-K_{i}(t-t_{m,i})}}$$

where *Q_i_* represents the thermal time-dependent potential fibre quality; *i* represents the different quality index, i.e. fibre length (mm), strength (cN tex^−1^) and micronaire. *Q*_max,*i*_ is the maximum value of a potential fibre quality index *i* of given cultivar; *K*_*i*_ is the relative growth rate (day^−1^), a constant that determines the curvature of the growth pattern; *t* is the physiological days and *t*_m_ is the inflection point at which the growth rate reaches its maximum value.

Under actual ambient conditions, growth will be less than the potential. Thus, the actual fibre quality is simulated by multiplying potential growth and reduction factors. The reduction factors are used to adjust actual growth rate according to the effect of temperatures, i.e. $f\;(\overset{-}{T}{)}_{i}$, the effect of daily mean temperature ($\overset{-}{T}$) on index *i*; *f*(*T*_min_)*_i_*, the effect of minimum temperature (*T*_min_) on index *i*; and *f*(TD)*_i_*, the effect of the difference between maximum (*T*_max_) and minimum temperatures (TD) on index *i*. Actual fibre quality of index *i*, Δ*Q*_a,*i*_, is then calculated as the product of the potential increment Δ*Q_i_* (Eq. (3)) and the reduction factors:

(3) $$\Delta Q_{a,i}=\Delta Q_{i}\times f\;(\overset{-}{T}{)}_{i}\times f\;(T_{\min}{)}_{i}\times f\;(\text{TD}{)}_{i}$$

For different fibre quality indices, the effect of reduction factors differed. Fibre length was only affected by daily mean temperature.

Fibre strength is the fibre's resistance to stretching, and is determined by fibre extension and duration of fibre thickening. Moreover, fibre strength is determined by environmental conditions and variety traits (Zhou *et al.* 2011). There is a significant positive correlation between cotton fibre strength and daily mean temperature during the formation of the fibre (Wang *et al.* 2009). Fibre strength increased linearly with daily mean temperature and decreased linearly with diurnal temperature difference between maximum and minimum (Ma *et al.* 2006).

Micronaire is a comprehensive fibre quality index that represents cotton fibre fineness and maturity (Rodgers *et al.* 2013). Micronaire increases with cotton fibre thickening (Bange *et al.* 2010). Daily minimum temperature greatly affects fibre micronaire (Ma *et al.* 2006). Higher daily minimum temperature could promote micronaire (Gipson and Ray 1969; Davidonis *et al.* 2004).

#### Model evaluation

To determine model performance, the root mean square error (RMSE) was used. RMSE quantifies the difference between observed and simulated results:

(4) $$\text{RMSE}=\sqrt{\frac{1}{N}\sum_{i=1}^{N}{\left(Y_{i}-X_{i}\right)}^{2}}$$

The relative error (RE) was used to determine the accuracy of the simulations:

(5) $$\text{RE}\;\;(\%)=\sqrt{\frac{1}{N}\sum_{i=1}^{N}{\left(\frac{Y_{i}-X_{i}}{X_{i}}\right)}^{2}}\times 100$$

where *Y_i_* and *X_i_* are the observed and simulated results, respectively. *N* is the number of observations. Data analysis was conducted using Microsoft Excel.

The effects of sowing dates, main stem and branch topping, and film cover on cotton fibre length, strength and micronaire in different scenarios were analysed using general linear model procedure (GLM) in SPSS 17 (IBM Corporation, New York, NY, USA).

### Simulations

To relate fibre quality to plant architecture, fibre length, strength and micronaire were expressed in relation to CPN (Fig. 3). Fruits of similar CPN can be regarded as being in the same cohort of fruits and therefore having experienced the same temperature regimes during their development. Moreover, the CPN is a useful measure for expressing the hierarchical position of a fruit with respect to the base of the plant, because the fruits at the same CPN number were formed around the same thermal time. This position affects the time of the formation of the fruit and its position in the canopy.

For the validation and scenario simulations, final fibre length, strength and micronaire values were averaged over the whole plant because the observed data at region levels were averaged. Simulations were run for 11 sites from 2006 to 2011 to validate the model performance by comparing with the nation-wide survey data. In each simulation run, 6 plants were simulated at a plant population density of 18.0 plants m^−2^ in the Xinjiang region, and 6.0 plants m^−2^ in the Yellow River and Yangtze River regions, in accordance with local cultivation practices. Topping means removal of apical buds with two phytomers. In Xinjiang, mulching using plastic film cover is widely used to increase soil temperature and protect the seedlings against low night-time temperatures. Mulching is not a standard practice in the other two regions. Hence, the effect of plastic film mulch on temperature was included in the simulations for Xinjiang only, and algorithms for the effect of film mulching are given by Zhang *et al.* (2008). Although film mulching does not directly affect fibre quality, it may modify fibre by an influence of plant growth and development by increasing soil temperature. Thus, the film mulching effects on fibre quality at different regions were also put into scenario treatments.

Since fibre quality is mainly affected by temperature, the fruit growing duration and spatial distribution of fruit are considered as major causes to the formation of fibre quality. Agronomy practices, e.g. sowing date, branch topping and film cover might have great influences on fibre quality. Thus, four scenarios for different sowing dates, main stem and branch topping dates, and film mulching were designed to explore their effect on fibre quality (Table 2). Simulations were run for three sites: Aksu in south Xinjiang (Xinjiang region), Huimin in Shandong (Yellow River region) and Yueyang in Hunan (Yangtze River region), for the years 1981–2010. Each simulation was run once using 6 plants for each year. The plant densities in the scenario studies were the same as in those used in model parameterization and validation.

**Table 2.** Simulation scenario design for exploring effects of sowing date, topping time and film cover on cotton fibre length, strength and micronaire. Values indicate number of days change in the variable compared with standard practice (0 days). ^a^Farmer's practice (0) for sowing, topping main stem and topping branches of cotton is on April 10, July 20 and August 5 in Xinjiang, April 25, July 30 and August 15 in Yellow River, April 10, August 10 and August 25 in Yangtze River regions, respectively. Except for the treatments, all other managements were set to farmer's practice in the studied regions. Film mulching is applied only in Xinjiang for different sowing dates and topping times, according to farmer's practice.

Scenario	Treatment	Practice day (event) comparing to farmer's tradition^a^ (days)					
Scenario 1	Sowing date	−20	−10	0	+10	+20	+30
Scenario 2	Topping main stem	−10	−5	0	+5	+10	+15
Scenario 3	Topping branches	0	+10	+20	+30		
Scenario 4	Film mulching	With film			Without film		

## Results

### Calibration of potential fibre quality increase rates

**Figure 5.**
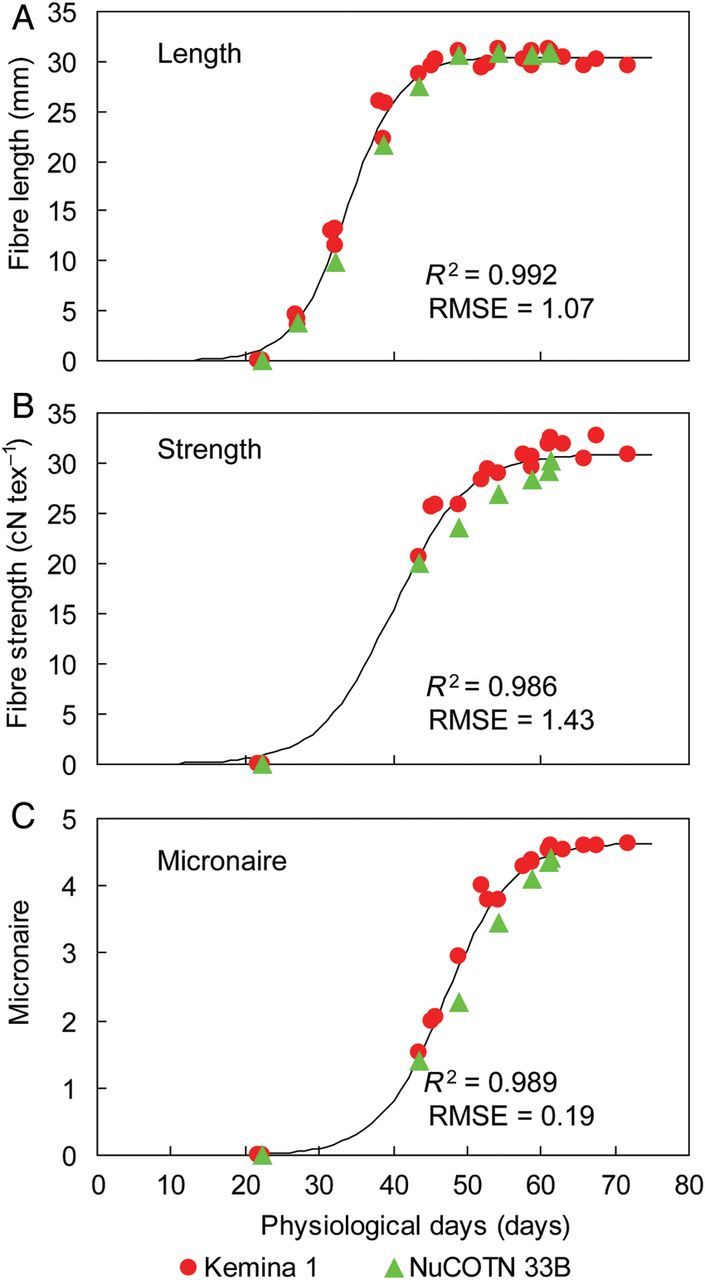
Calibration of potential fibre length (A), strength (B), micronaire (C) against fruit age (days after squaring) in cotton in Anyang in 2007. Symbols of different shape and colour indicate different cultivars.

Parameter values were obtained through fitting the relationship of potential quality indices, i.e. length, strength and micronaire, and showed a good fitness with observed values under unlimited water and N conditions (Fig. 5). The two testing cultivars showed no difference. The *R*^2^ of curve fitting ranged from 0.992 to 0.989, the RMSE between estimated and observed values was 1.07 mm for length, 1.43 cN tex^−1^ for strength and 0.19 for micronaire. The time that a fruit reached its maximum increase rate for length was at 34 physiological days after squaring (fruit age), for strength at 40 days and for micronaire at 47 days (Table 3).

**Table 3.** Parameters estimated from the nonlinear regression for potential cotton fibre length, strength and micronaire in 2007 in Anyang, China. ^a^The days indicate the age of a fruit after the emergence of the square, expressed in physiological days (days required under optimal temperature). The values are estimated mean ± SE.

Quality index (*Q_i_*)	*Q*_max,*i*_	*K_i_* (day^−1^)	*t*_m,*i*_ (days)^a^	*R*^2^	RMSE
Length (mm)	30.47 ± 0.26	0.28 ± 0.015	33.78 ± 0.23	0.992	1.07
Strength (cN tex^−1^)	30.94 ± 0.53	0.23 ± 0.032	39.97 ± 0.85	0.986	1.43
Micronaire	4.648 ± 0.09	0.22 ± 0.020	47.03 ± 0.34	0.989	0.19

### Effect of temperature on fibre quality

Quadratic regression between mean temperature and effect on fibre length $f\;(\overset{-}{T}{)}_{L}$ is given in Eq. (6), *R*^2^ = 0.67. Effect of mean temperature on fibre length had a maximum of 1 near the optimum temperature of 25 °C, and zero at the lower and upper temperature thresholds for fibre growth of 9 and 42 °C, respectively:

(6) $$f\;(\overset{-}{T}{)}_{L}=-0.0038{\overset{-}{T}}^{2}+0.1904\overset{-}{T}-1.3694$$

The effect of mean temperature on fibre strength ($f\;(\overset{-}{T}{)}_{S}$, Eq. (7)) ranged between 0 and 1. Parameter values were obtained through fitting experimental data, *R*^2^ = 0.55. When daily average temperature was over 30 °C, the value equalled 1:

(7) $$f\;(\overset{-}{T}{)}_{S}=0.0198\overset{-}{T}+0.4657$$

The effect of the difference between minimum and maximum daily temperature on fibre strength (*f*(TD)_S_, Eq. (8)) was calculated as a linear relationship, and *R*^2^ of the fitted equation was 0.56:

(8) $$f\;(\text{TD}{)}_{S}=-0.0216(T_{\max}-T_{\min})+1.136$$

Micronaire was only affected by daily minimum temperature. The effect of minimum temperature on fibre micronaire (*f*(*T*_min_)_M_, Eq. (9)) was described as a linear regression and *R*^2^ of the fitted equation was 0.67. When minimum temperature was over 25 °C, the value of the reduction factor was 1:

(9) $$f\;(T_{\min}{)}_{M}=0.0244T_{\min}+0.3874$$

### Simulations of cotton and fibre growth

**Figure 6.**
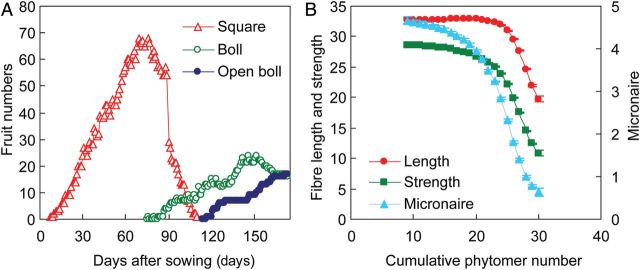
Simulated dynamic course of fruit numbers (squares, bolls and open bolls) (A) and final cotton fibre length (mm), strength (cN tex^−1^) and micronaire against CPN (phytomer position counted from the bottom of the plant) (B) at 210 simulated days after sowing (*n* = 10, error bars show 2 × SE). The model was run for monoculture of cotton with MC application at a plant population density of 3.6 plants m^−2^, and main shoot and branch topping applied at 30 July and 30 August, respectively, to terminate further appearance of new phytomers.

Fruits at CPN values lower than 20 showed comparable values for all three aspects of fibre quality, although micronaire showed a decreasing trend with increasing CPN over the whole range of CPN values (Fig. 6). Fruits that developed at high ranks, especially above a CPN value of 25, showed very low fibre quality in every aspect.

### Model validation

**Figure 7.**
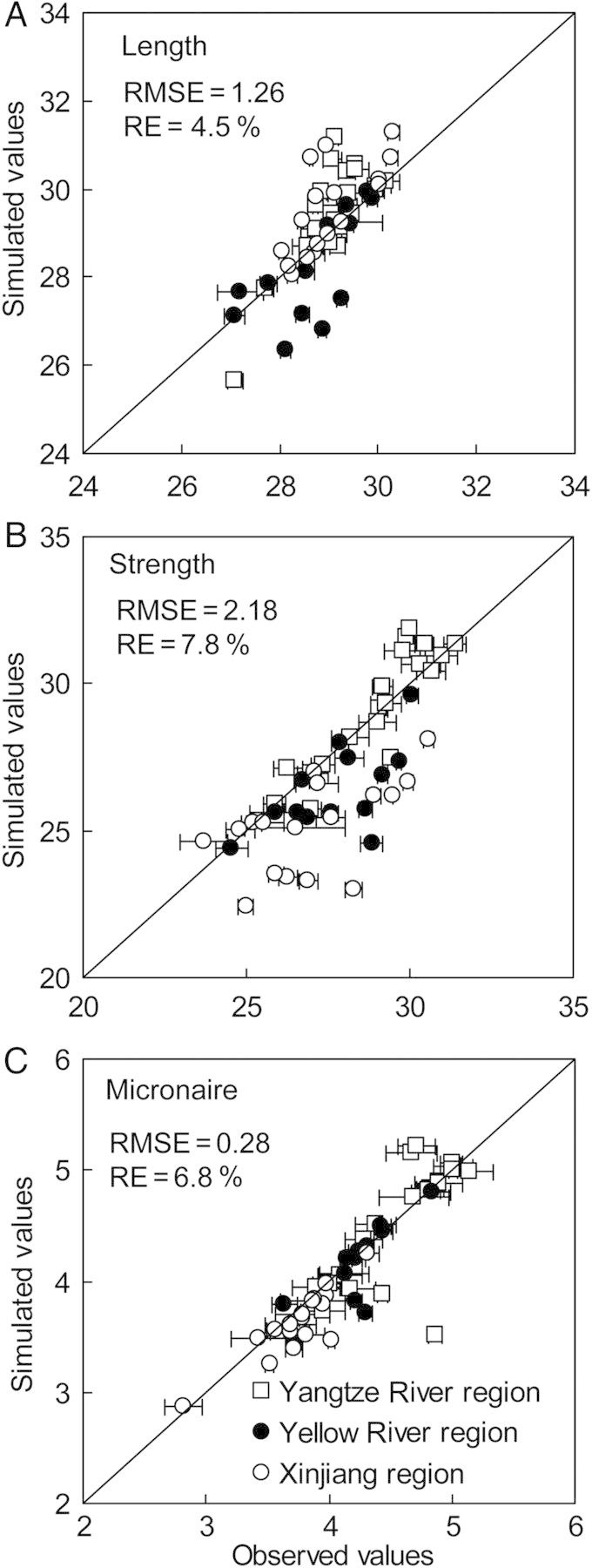
Comparison of observed and simulated cotton fibre length (A), strength (B) and micronaire (C) from 2006 to 2011 for 11 sites in major cotton producing regions in China. In the simulations, sowing dates were 10 April, 25 April and 15 April in Xinjiang, Yellow River and Yangtze River regions, respectively. The time of main stem topping was 10 July in the north part and 20 July in the south part of Xinjiang region, and 30 July in Yellow River and Yangtze River regions. Branch topping time was 15 days after topping of the main stem in all cases.

There was overall good correspondence between simulated and observed fibre length (Fig. 7A), strength (Fig. 7B) and micronaire (Fig. 7C). The RMSE between simulated and observed values for fibre length, strength and micronaire was 1.26 mm, 2.18 cN tex^−1^ and 0.28, respectively. The RE (%) for model accuracy between the simulated and observed values for fibre length, strength and micronaire was 4.5, 7.8 and 6.8 %, respectively. These relatively small errors indicate that credible and useful results can be obtained with CottonXL for the conditions the model was calibrated for.

### Scenarios studies

#### Sowing date

**Figure 8.**
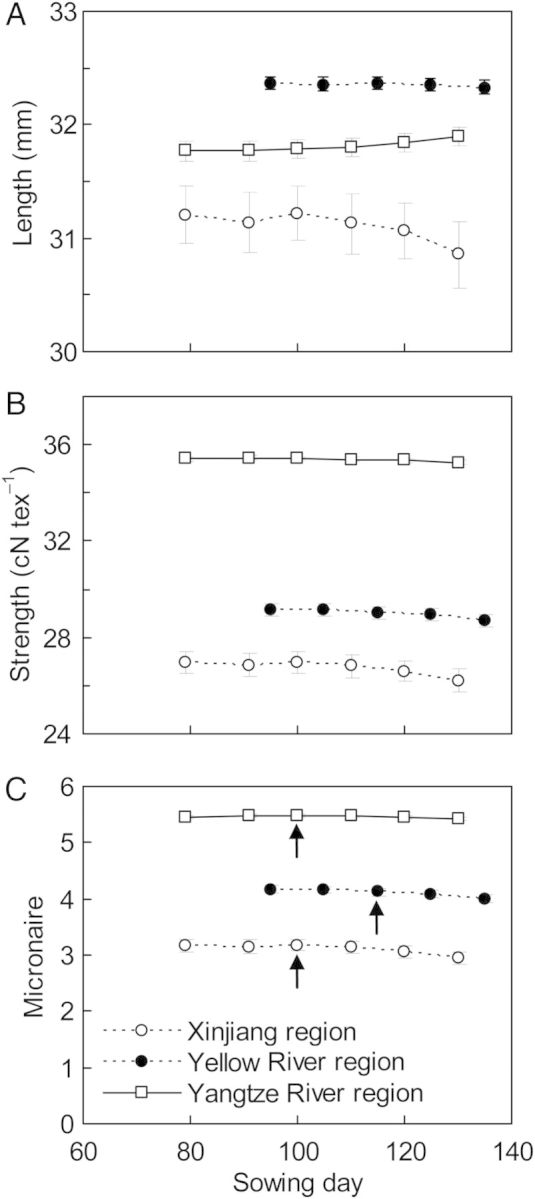
Simulated cotton fibre length (A), strength (B) and micronaire (C) against sowing date for three sites located in northwest (Aksu), Yellow River (Huimin) and Yangtze River (Yueyang) cotton producing regions in China averaged over the period 1981–2010. The arrow points to traditional sowing dates.

Delaying the sowing date significantly (*P* > 0.01) increased fibre length in the Yangtze River valley, but significantly (*P* < 0.05) decreased fibre length in the Xinjiang when sowing date was postponed by 20 days. It had no significant (*P* = 0.225) influence on fibre length in the Yellow River valley. Over 30 years and three regions, cotton fibre strength was not significantly (*P* = 0.393) decreased by delaying sowing date; however, fibre micronaire was significantly (*P* = 0.023) decreased. Here, the variation of fibre length (as represented by the size of the error bars in Fig. 8) due to climate variation (i.e. the crop experiencing a different climate over the years) was much greater compared with the other regions. The distribution of fibre indices: length, strength and micronaire, presented as the frequency of fruit numbers against the value of each fibre quality index, was not significantly (*P* > 0.05) affected by sowing date, except for a significant (*P* < 0.01) increase in the percentage of fruits with low micronaire values (2.25–3.75) at 30 days delay of sowing date (5 August) in Xinjiang **[see Supporting Information]**. The results from scenario analysis based on regional temperature for cotton fibre quality showed good agreement with the actual distribution of fibre quality (Fig. 1).

#### Topping

**Figure 9.**
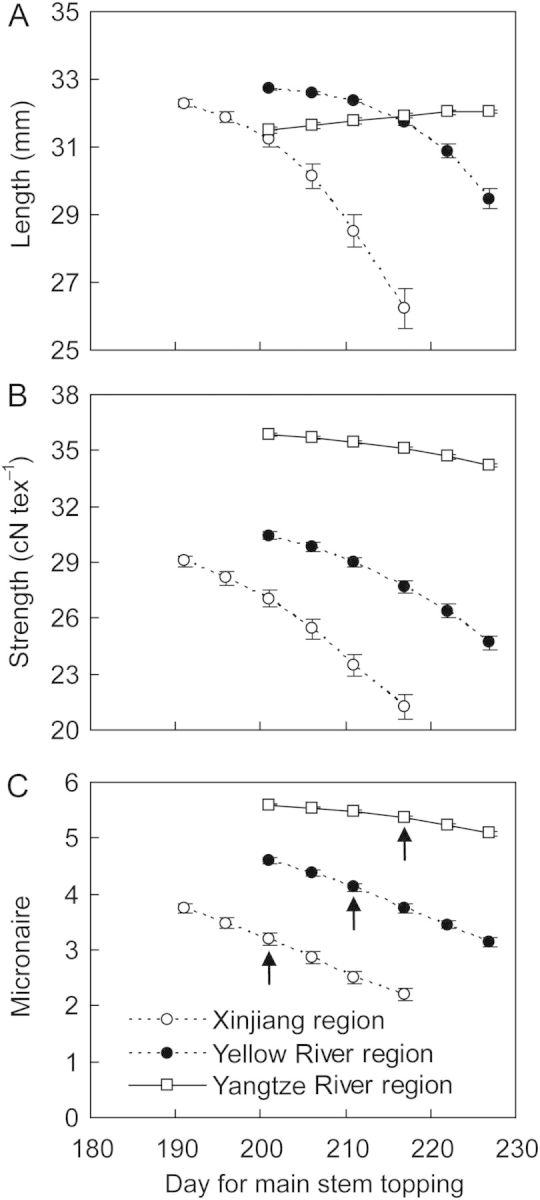
Simulation results for cotton fibre length (A), strength (B) and micronaire (C) affected by main stem topping for three sites located in northwest region (Aksu), Yellow River valley (Huimin) and Yangtze River valley (Yueyang) cotton producing regions in China averaged over the period 1981–2010. The arrow points to traditional topping time.

**Figure 10.**
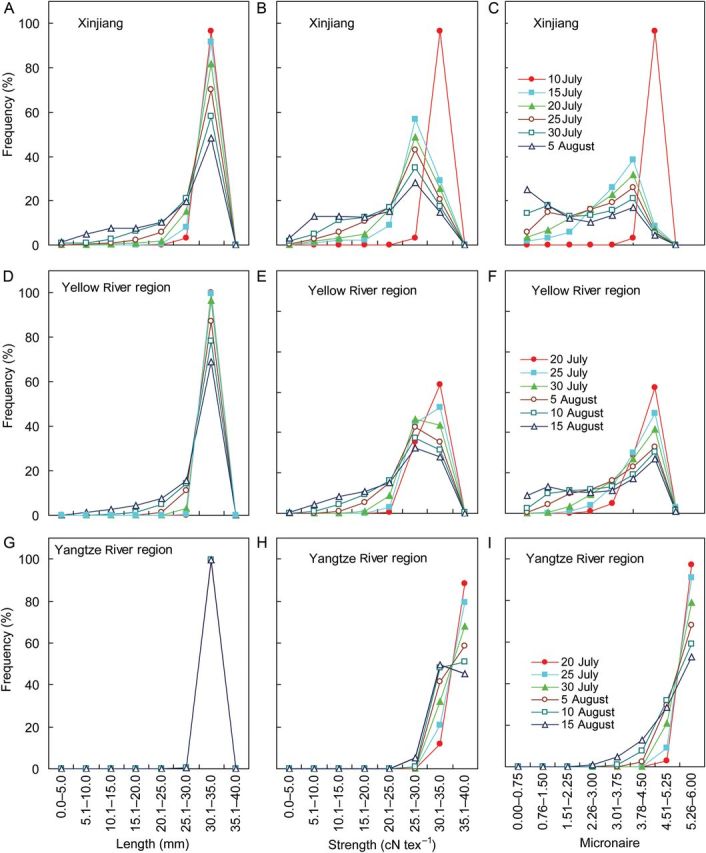
Frequency of fruit numbers for different aspects of fibre length, strength and micronaire affected by main stem topping time (legend) over the period 1981–2010 at three cotton producing regions in China.

The times of main stem and branch topping determine the final number of fruits on a plant. Early topping practice resulted in all the fruits of high quality. Over 30 years and three sites, simulated fibre length, strength and micronaire were significantly (*P* < 0.01) decreased with later timing of main stem topping; however, delaying topping main stem time significantly increased fibre length in the warm and humid region of Yangtze River (Fig. 9). This effect was greatest for the Xinjiang region and the Yellow River region. The effect of topping was smallest in the Yangtze River region. The percentage of fruits (frequency) of high quality was greatly affected by the time of main stem topping (Fig. 10). As topping time was delayed, the number of fruits with high length, strength and micronaire decreased significantly (*P* < 0.01). Early topping of main stems significantly (*P* < 0.01) increased the frequency of fruits with longer fibre length (30–35 mm) in the Xinjiang and Yellow River regions, but there was no effect on the frequency distribution of fibre length in the Yangtze River region. Earliest time for topping main stem (10 days earlier than traditional time) significantly (*P* < 0.001) increased the percentage of fruits with higher fibre strength (30–35 cN tex^−1^) and micronaire (4.5–5.25), especially in Xinjiang (Fig. 10). Delaying topping time (10–15 days) significantly (*P* < 0.01) increased the percentage of fruits with lower length, strength and micronaire (Fig. 10) in all regions, thus the overall fibre qualities were significantly (*P* < 0.05) decreased.

**Figure 11.**
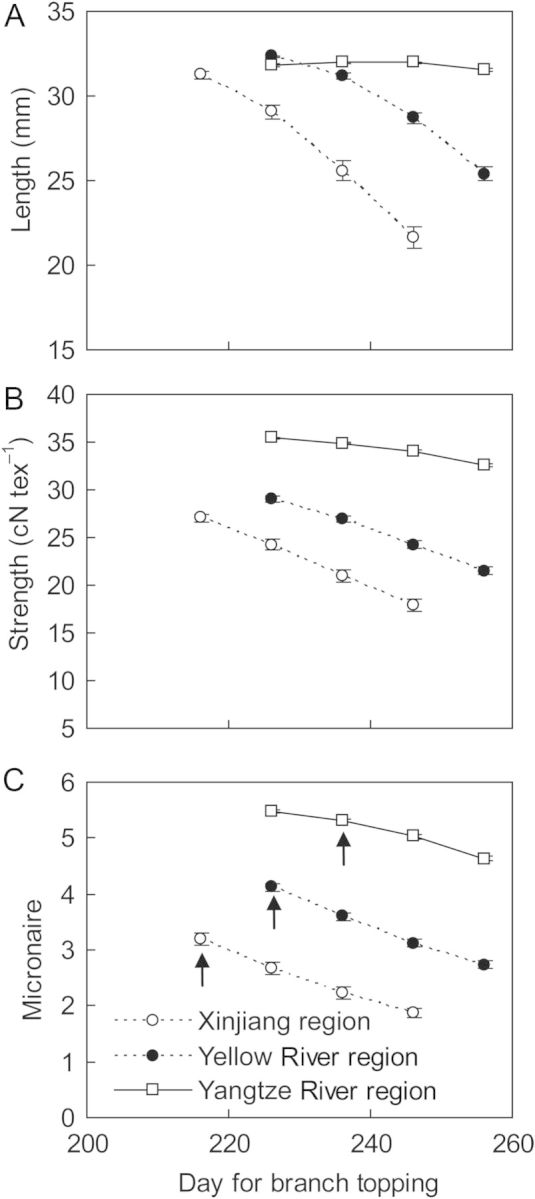
Simulation results for cotton fibre length (A), strength (B) and micronaire (C) affected by branch topping for three sites located in northwest (Aksu), Yellow River (Huimin) and Yangtze River (Yueyang) cotton producing regions in China averaged over the period 1981–2010. The arrow points to traditional branch topping time.

Delaying branch topping time resulted in a significant (*P* < 0.001) decrease in fibre length, strength and micronaire in all regions simulated (Fig. 11), especially in Xinjiang and the Yellow River region. Delaying branch topping time also caused a significantly (*P* < 0.001) high distribution frequency of fruits with low fibre length, strength and micronaire. Current time for branch topping (5 August in Xinjiang, 15 August in Yellow and Yangtze River regions) showed a significantly higher percentage of fruits with high length, strength and micronaire than delayed topping branch time, except for a lower value in length in the warmer Yangtze River region **[see Supporting Information]**.

#### Film mulching

The results of Scenario 4 (with and without film mulching) at the three regions did not show a significant effect on fibre length (*P* = 0.794), strength (*P* = 0.570) and micronaire (*P* = 0.559), and also did not affect the distribution of fruit frequency in fibre length, strength and micronaire. However, applying film cover in the Yellow River region slightly decreased the percentage of fruits with a high strength (25.0–35.0 cN tex^−1^) value **[see Supporting Information]**. Although film mulching plays an important role in improving cotton yield in Xinjiang, its effect on fibre quality and its distribution is less important when a proper topping time is practiced according to the heat resources available.

## Discussion

The extended version of CottonXL presented here gave good predictions of fibre length, strength and micronaire in relation to boll age and prevailing temperatures during boll development. Model output corresponded well with independent experimental data. The observation that fibre quality decreases severely for fruits of a high CPN, was consistent with results from previous studies (Davidonis *et al.* 2004; Zhou *et al.* 2011), showing late-formed fruits had rather low fibre quality due to low temperatures. Fibre strength was underestimated by the model in the Yellow River and Xinjiang regions. This was probably due to water shortage which often occurs in these regions, whereas the model was calibrated for optimal water supply. Water shortage results in stronger fibres, but the current version of CottonXL does not account for this effect. The advantage of this model over the model of Zhao *et al.* (2012) was the calculation of fibre quality model at individual single boll level and the upscaling to plant and crop level, which made analysis of effects of regional conditions on fibre quality possible.

### Controlling the effect of temperature by sowing date and mulching

Sowing date did not significantly change fibre quality in the Yellow and Yangtze River regions. However, in Xinjiang, where the temperature is relatively low during the early cotton growing period, delay of sowing date decreased fibre quality because low temperatures at the end of growing period could prevent bolls from reaching full maturity. This effect was most prominent for fibre length which also showed considerable variation over the years (1981–2010) due to the effects of changing weather conditions. Later sowing negatively impacts cotton yield (Cathey and Meredith 1988; Bozbek *et al.* 2006), but does not influence boll numbers (Barradas and Lopez-Bellido 2009). Therefore, breeding strategies that provide low-temperature tolerance might increase flexibility for planting times especially in cooler regions (Tan *et al.* 2011). We conclude that the effect of sowing date on cotton yield and quality depends on heat resource availability. Film mulching could increase soil temperature, soil moisture content and yield (Dong *et al.* 2008), and therefore it could play an important role in improving yields in the Xinjiang region. The limitation for heat resource can only be overcome by film mulching at the beginning, when the sun can reach the soil, and generate very high temperatures under the plastic and in the soil. Our simulations found no significant effect of film mulching on fibre quality for this region because the film effects mainly occurred at an early stage and appearance of late fruits which have no sufficient heat to be matured was stopped by topping, although it is considered a useful agronomic practice to enhance cotton yield (Dong *et al.* 2008).

### Main stem and branch topping

For optimizing topping time, farmers in China usually use the number of fruit branches as an indicator while the number of nodes above the first white flower is used in the USA (Bynum and Cothren 2008). Our simulation of delaying main shoot topping, which results in a greater number of fruit branches and fruits on the plant, predicted a substantially reduced cotton fibre quality due to the increase of late-formed bolls. This was especially the case in Xinjiang and Yellow River valley cotton producing regions, whereas it did not greatly affect fibre quality in the Yangtze River valley due to the longer growing season. Topping earlier than the reference time greatly increases fibre quality especially in the Xinjiang region, but also leads to a reduction of total boll number and yield due to removal of new fruiting branch sites on the plant (Teague *et al.* 2011). Increasing population density and application of MC could improve cotton fibre quality (Ren *et al.* 2013). According to our simulations, combining high density and MC with early topping would be practical, since the late-formed fruits that may not be mature at the time of harvest will be prevented from appearing (Siddique *et al.* 2002; Ren *et al.* 2013).

Fruits that are initiated <60–70 calendar days before final harvesting do not contribute to yield (Zhang *et al.* 2008). Therefore, proper timing of topping or other measures to prevent useless fruits from forming is important for increasing yield and quality of the yield. From our scenario analysis, we conclude that delaying branch topping after main stem topping would greatly decrease fibre quality, especially in the Yellow River valley and Xinjiang regions. Delaying branch topping to 25 days after main stem topping decreased fibre quality by 9 % (length: 11 %, strength: 6 %, micronaire: 11 %) compared with 15 days after main stem topping. Branch topping 15–25 days after main shoot topping is a common practice for Chinese farmers in order to increase yields at the expense of fibre quality. Our simulation results showed that branch topping should be done at same time or no later than 15 days after main shoot topping, otherwise, acceptable fibre quality is unlikely to be reached.

## Conclusions

CottonXL was capable of simulating cotton fibre quality under various conditions such as climate, cultivar, sowing date and topping time, by taking into account the variation in temperature regimes experienced by the developing fruits. The model is therefore a useful tool to explore cotton fibre quality, and is especially suitable to explicitly predict the variation of quality aspects within a plant as a result of differential periods of development of the fruits. Also it can be used to optimize plant architecture in order to get high fibre quality in given environmental conditions and management scenarios by manipulating plant architecture in the model, as well as to help farmers understand how the plant architecture determines crop development and quality. Since cotton fibre quality can be improved by a proper combination of MC application and population density (Ren *et al.* 2013), optimal management taking into account density, MC, sowing date and topping times for both high yield and quality can be explored and predicted using the current model.

## Sources of Funding

This research was supported by ‘948’ Program (2011-G19), Transgenic major project (2012ZX08013010), the program of the Modern Agricultural Industry Technology System (CARS-18-18) and Chinese Special Fund for Meteorological Research in the Public Interest (GYHY201206047).

## Contributions by the Authors

L.Z., Z.L., X.P. and X.W. conceived and designed the experiments; L.M., X.W., X.Z. and S.W. performed the experiments; X.W. and L.Z. analysed the data; L.Z. and J.E. developed the model; and X.W., L.Z., J.E. and W.W. wrote the paper.

## Conflicts of Interest Statement

None declared.

## Supporting Information

The following Supporting Information is available in the online version of this article –

**Fig. 1.** Dynamic courses of minimum and maximum temperatures at studied sites. Data for the Anyang site are given for the year 2007. Values for other sites are based on averages from 2006 to 2011.

**Fig. 2.** Frequency of fruit numbers for different aspects of fibre length, strength and micronaire affected by sowing date (legend) over the period 1981–2010 at three cotton producing regions in China.

**Fig. 3.** Frequency of fruit numbers for different aspects of fibre length, strength and micronaire affected by the time of topping branches (legend) over the period 1981–2010 at three cotton producing regions in China.

**Fig. 4.** Frequency of fruit numbers for different aspects of fibre length, strength and micronaire affected by film mulching over the period 1981–2010 at three cotton producing regions in China.

Additional Information
