# Supplementary material for: Predicting the effects of environment and management on cotton fibre growth and quality: a functional–structural plant modelling approach
Source: AoB Plants. 2014 Jul 9;6:plu040. doi: 10.1093/aobpla/plu040 (PMC4224667; doi:10.1093/aobpla/plu040)
Supplement: Additional Information [file supp_6_plu040_index.html]

Predicting the effects of environment and management on cotton fiber growth and quality: a functional-structural plant modelling approach — Predicting the effects of environment and management on cotton fibre growth and quality: a functional–structural plant modelling approach — Additional Information 

# Predicting the effects of environment and management on cotton fibre growth and quality: a functional–structural plant modelling approach

## Additional Information

Additional Information

**Files in this Data Supplement:**

- Additional Information - ppt file
