# Supplementary material for: Predicting the effects of environment and management on cotton fibre growth and quality: a functional–structural plant modelling approach
Source: AoB Plants. 2014 Jul 9;6:plu040. doi: 10.1093/aobpla/plu040 (PMC4224667; doi:10.1093/aobpla/plu040)
Supplement: Additional Information [file supp_plu040_plu040supp.ppt]

## Slide 1
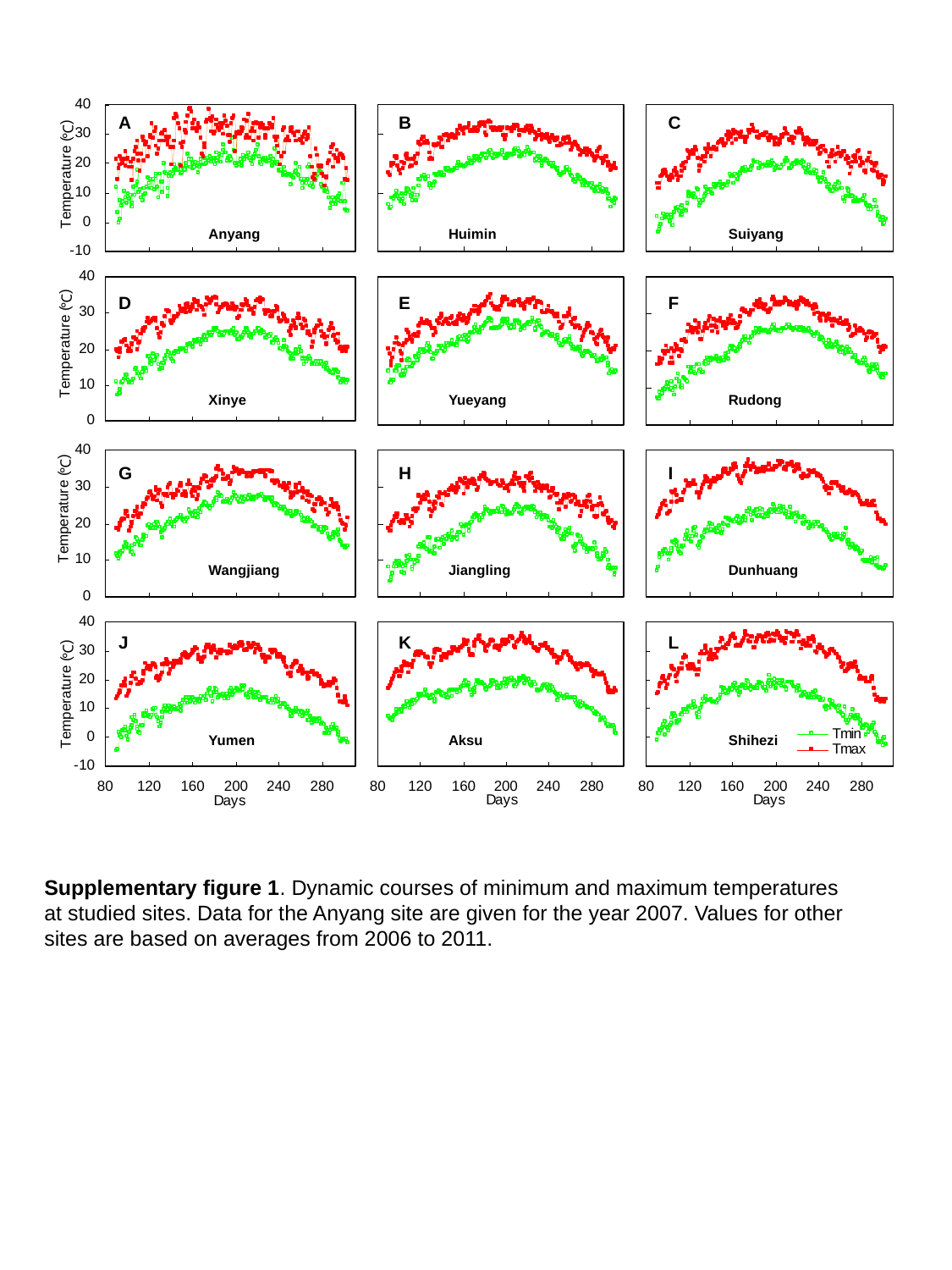

A
B
C
Anyang
Huimin
Suiyang
D
E
F
Xinye
Yueyang
Rudong
G
H
I
Wangjiang
Jiangling
Dunhuang
J
K
L
Yumen
Aksu
Shihezi
Supplementary figure 1. Dynamic courses of minimum and maximum temperatures at studied sites. Data for the Anyang site are given for the year 2007. Values for other sites are based on averages from 2006 to 2011.

## Slide 2
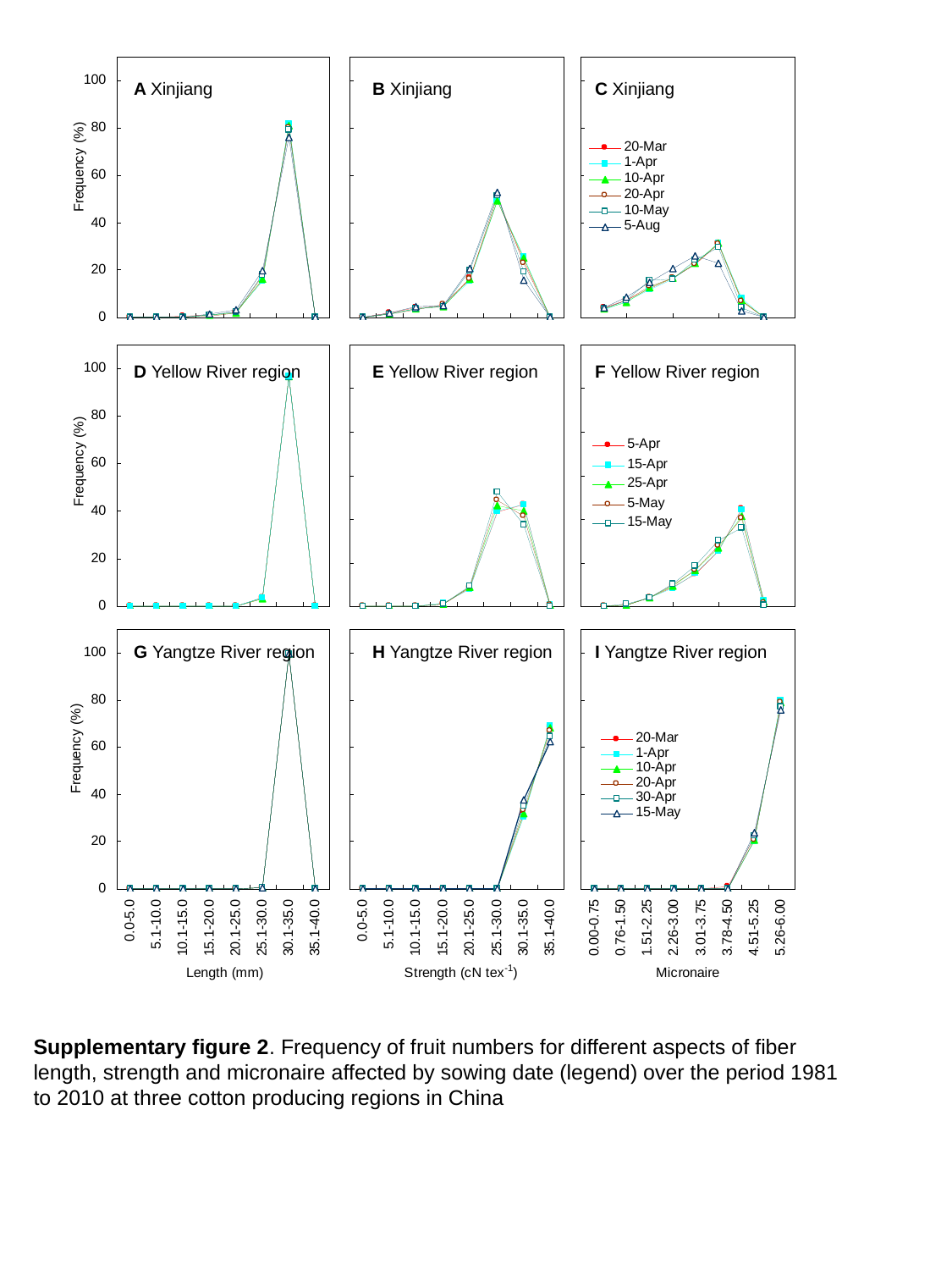

A Xinjiang
B Xinjiang
C Xinjiang
D Yellow River region
E Yellow River region
F Yellow River region
G Yangtze River region
H Yangtze River region
I Yangtze River region
Supplementary figure 2. Frequency of fruit numbers for different aspects of fiber length, strength and micronaire affected by sowing date (legend) over the period 1981 to 2010 at three cotton producing regions in China

## Slide 3
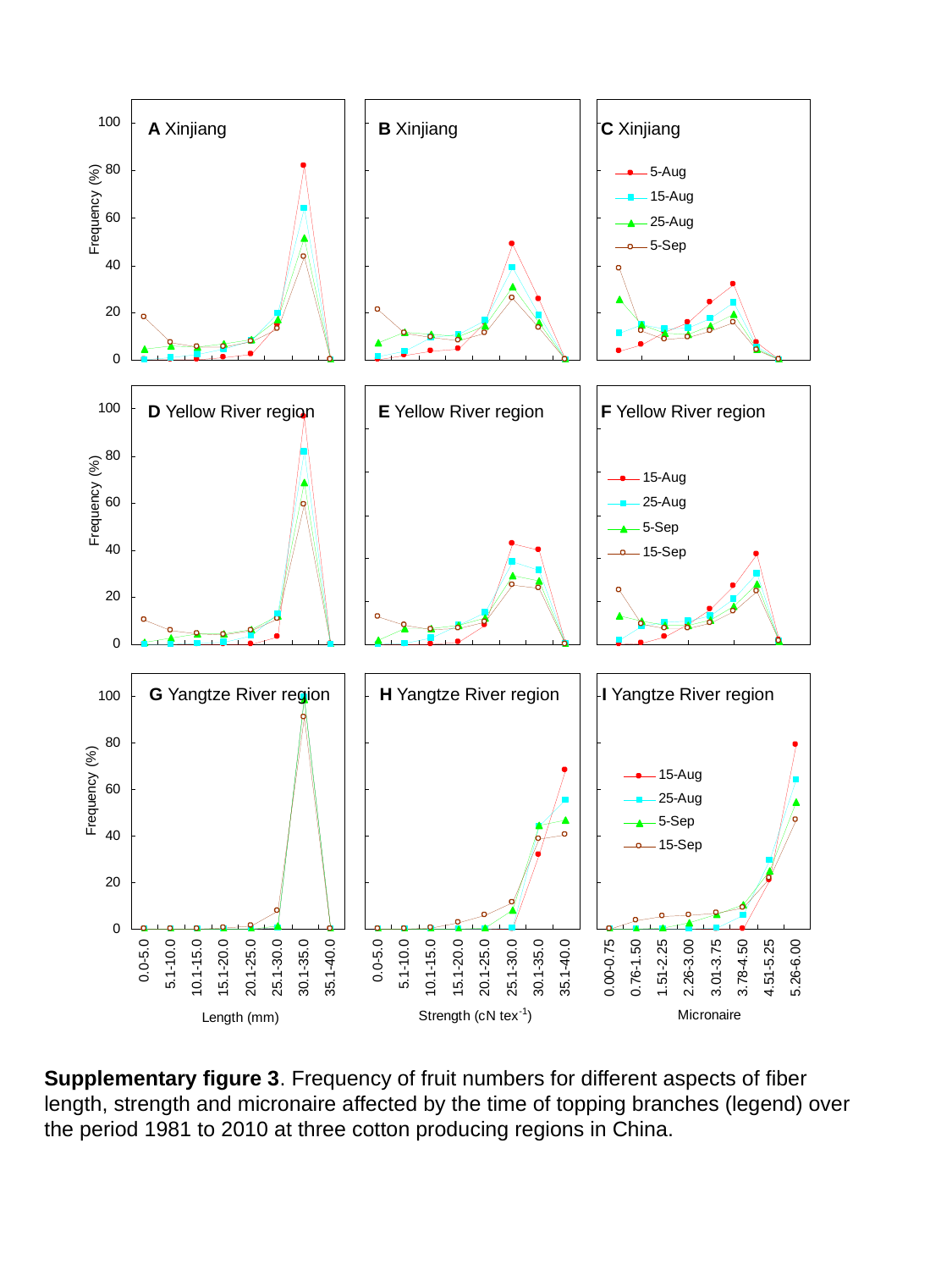

A Xinjiang
B Xinjiang
C Xinjiang
D Yellow River region
E Yellow River region
F Yellow River region
G Yangtze River region
H Yangtze River region
I Yangtze River region
Supplementary figure 3. Frequency of fruit numbers for different aspects of fiber length, strength and micronaire affected by the time of topping branches (legend) over the period 1981 to 2010 at three cotton producing regions in China.

## Slide 4
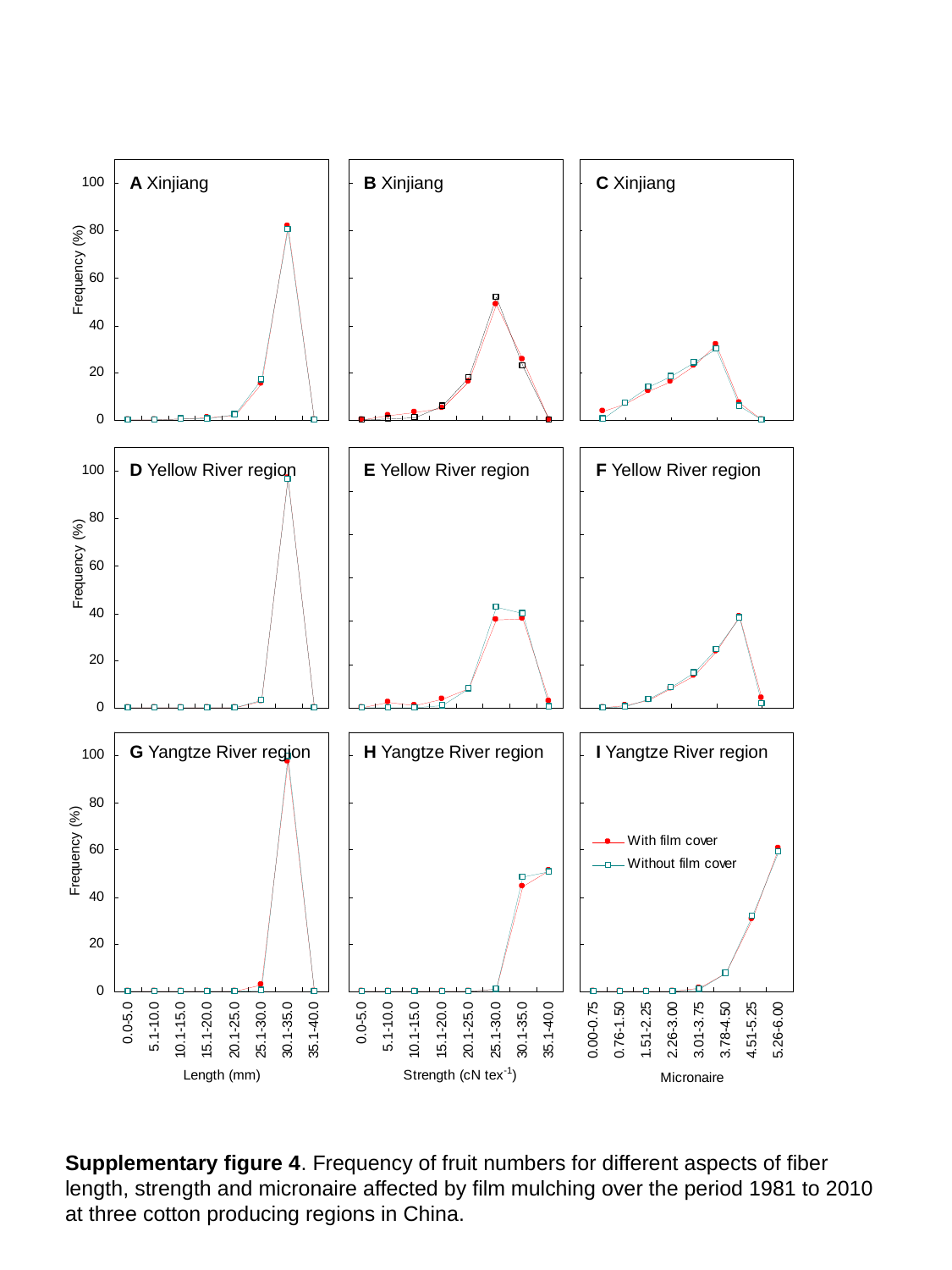

A Xinjiang
B Xinjiang
C Xinjiang
D Yellow River region
E Yellow River region
F Yellow River region
G Yangtze River region
H Yangtze River region
I Yangtze River region
Supplementary figure 4. Frequency of fruit numbers for different aspects of fiber length, strength and micronaire affected by film mulching over the period 1981 to 2010 at three cotton producing regions in China.
